# Volatile organic breath components and exercise induced bronchoconstriction in asthmatic children

**DOI:** 10.1186/s13223-021-00622-6

**Published:** 2021-11-27

**Authors:** M. R. van der Kamp, J. M. M. Driessen, M. P. van der Schee, B. J. Thio, F. H. C. de Jongh

**Affiliations:** 1 Department of Paediatrics, Medisch Spectrum Twente, Koningsplein 1, 7512 KZ Enschede, Netherlands; 2 Department of Biomedical Signals and Systems, University of Twente, Enschede, Netherlands; 3 Research and Development, Owlstone Medical, Cambridge, UK; 4 Department of Pulmonology, Medisch Spectrum Twente, Enschede, Netherlands; 5 Department of Engineering Fluid Dynamics, University of Twente, Enschede, Netherlands

**Keywords:** Asthma, Children, Breath condensate, Exercise induced bronchoconstriction, Salbutamol

## Abstract

**Introduction:**

Asthma is one of the most common chronic diseases in childhood and is generally characterized by exercise induced bronchoconstriction (EIB). Assessing EIB is time consuming and expensive as it requires a fully equipped pulmonary function laboratory. Analysis of volatile organic compounds (VOCs) in breath is a novel technique for examining biomarkers which may associate with asthma features. The aim of this pilot study was to identify potential markers in the relationship between EIB and VOCs.

**Methods:**

Children between four and 14 years old were asked to provide a breath sample prior to undergoing an exercise challenge test to assess for EIB.

**Results:**

Breath samples were collected and analyzed in 46 asthmatic children, 21 with EIB and 25 without EIB (NO-EIB). Molecular features (MFs) were not significantly different between EIB and NO-EIB controls. 29 of the 46 children were corticosteroid naïve, 10 with EIB and 13 without. In the corticosteroid naïve group EIB was associated with increased MF23 and MF14 in the lower breath sample (p-value < 0.05).

**Conclusion:**

This pilot study shows that EIB was related to an increased MF14 and MF23 in corticosteroid naïve children. The tentative identities of these compounds are octanal and dodecane/tetradecane respectively. These candidate biomarkers have a potential to enable non-invasive diagnosis of EIB in steroid-naïve children.

*Trial registration* This study is registered in the Netherlands trial register (trial no. NL6087) at 14 February 2017.

## Introduction

Asthma is one of the most common chronic diseases in childhood and is generally characterized by exercise induced bronchoconstriction (EIB) [1]. EIB has a detrimental effect on quality of life and athletic performance [2]. Evaporation of water content of the airway lining during exercise-induced hyperpnea leads to changes in osmolarity of the airway wall [3]. These changes cause mast cells, residing in the inflamed asthmatic airway wall, to release mediator compounds [4]. These mediators, including prostaglandin D4, leukotriene D4 and histamine, cause contraction of smooth muscle cells, increase in mucus production and oedema of the epithelium. An increase in metabolites associated with these mediators has already been found in urine directly after an episode of EIB [5].

Analyzing EIB using indirect bronchial provocation testing may be experienced as obtrusive by children and can be time consuming and expensive, as they require a fully equipped pulmonary function laboratory with experienced staff [6, 7]. Analysis of volatile organic compounds (VOCs) in breath is a novel non-obtrusive technique for examining endogenous and exogenous biomarkers (prescribed drugs, diet and environmental exposure) [8]. Its potential association with asthma phenotypes makes VOCs in breath a current topic of interest and suggests to explore whether it may differentiate children with non-specific respiratory symptoms from true asthmatics, and predict steroid responsiveness in the asthmatics [8]. Evidence is emerging how these compounds originate from airway inflammatory cells through processes such as oxidative stress [9]. However, no studies have yet investigated the use of breath VOC biomarkers in relation to the occurrence of EIB in children.

The aim of this pilot study was to identify potential biomarkers for EIB in children with asthma.

## Methods

### Study design

This cohort study had a prospective cross-sectional design. Children with asthma, who participated in the WEARCON trial [10], were asked to provide a breath sample prior to undergoing an exercise challenge test to assess for EIB. This study was approved by the medical ethics committee of the Medical Spectrum Twente and was registered in the Netherlands trial register (trial no. NL6087).

### Sample size and population

The population consisted of 46 pediatrician diagnosed asthmatic children aged between 4 and 14 years old with respiratory symptoms. All participants underwent an exercise challenge test (ECT) in combination with pulmonary function tests (PFT) to determine if they had EIB. The ECT was performed in a climate chamber (10 °C) according to the American Thoracic Society guidelines [6, 11]. Children aged 8–14 years old performed the ECT on a treadmill for 6 min with submaximal exercise load (steady-state heart rate of 85% of the maximal heart rate (220—age)) and their nose clipped. The inclination of the treadmill was 10%. Children aged 4–7 years old performed the exercise on a jumping castle for 6 min as described by van Leeuwen et al. [12]. EIB was defined as a post-exercise maximal decrease in forced expiratory volume in 1 s (FEV_1_) of ≥ 13% (if on inhaled corticosteroids(ICS)) or ≥ 20% (if not on ICS) [13]. To prevent the influence of the direct pharmacological effect of medication, subjects were asked to withhold their medication before the ECT according to the withholding times stated in the ERS standard of bronchial challenge testing [14].

Demographic characteristics (gender, age and body mass index) of the subjects were retrieved from electronic patient records. Other clinical metadata involved the dichotomous variable ICS use (orally confirmed adherent use of at least 100 µg ICS twice daily), the baseline lung function (FEV_1_% predicted) and the maximal post-exercise fall in FEV_1_ during the ECT. Moreover, allergies were assessed in the subjects with a radioallergosorbent test (RAST) within the last three years before the ECT. Subjects with an IgE level > 0.35 kU/L for at least one of the inhalation allergies (house dust mites, grass pollen, tree pollen, cat epithelium, dog epithelium, fungi and herb pollen) were classified as allergic. A subset of our population was on ICS, which strongly modifies asthmatic airway inflammation. To explore this in more detail an additional analysis was performed with stratification based on ICS usage in the children.

### Breath sample collection & analysis

Breath samples were collected immediately prior to the ECT using a ReCIVA® Breath Sampler (Owlstone Medical Ltd.). ReCIVA is capable of simultaneous collection of up to two breath fractions. The device contains two independent pumps each attached to two of the sorbent tubes in the Breath Biopsy Cartridge. Each pump can be activated separately at different points in breath collection to capture air from pre-determined breath fractions.

ReCIVA collects VOCs from exhaled breath directly onto four Tenax TA/Carbograph 5TD sorbent tubes integrated into the Breath Biopsy® Cartridge (Owlstone Medical) To minimize background VOCs originating from ambient air, subjects inhale air through ReCIVA that has been filtered. ReCIVA includes a non-return valve that ensures inhaled air passes through CASPER and exhaled air is released directly to the environment after VOC extraction.

### Collection

ReCIVA collects breath from subjects during regular tidal breathing, requiring no specialist breathing maneuvers. During collection, the system initially learns a subject’s breathing pattern allowing it to control pump activation to specifically capture selected breath fractions. In this study one pump collected air from the upper airways while the other collected breath enriched for the alveolar fraction. The upper airway fraction is more likely to contain VOCs originating directly in the bronchial mucosa and epithelium. Deeper fractions include alveolar air which exchanges organic compounds with the bloodstream. Blood carries many VOCs originating throughout the body, which are associated with a wide range of biochemical and metabolic processes including inflammation. In this study, 500 mL of breath was collected onto each sorbent tube, meaning a total of 1 L was samples from each breath fraction.

### Sample analysis

Samples were analysed using the Breath Biopsy platform in the Breath Biopsy Laboratory (Owlstone Medical Ltd.). Samples were dry purged to remove excess water and desorbed using a TD100-xr thermal desorption autosampler (Markes International) and transferred onto a VF-5 ms column (60 m × 0.25 mm × 0.25 um; CP8960 Agilent Technologies) using 1:2 split injection. Chromatographic separation was achieved via a programmed method (50–310 ℃ in 60.3 min. at 2.0 mL/min.) on a 1310 oven (Thermo Fisher Scientific) and mass spectral data acquired using an electron impact ionization time-of-flight (EI-TOF) BenchTOF HD mass spectrometer (Markes International).

### Data analysis

#### Feature extraction

Features list were extracted from TD-GC-MS chromatograms for statistical analysis. Feature extraction involves identifying a set of characteristics indicative of a compound and aligning them across all samples to ensure that the same feature is consistently identified and extracted when present in any sample from the dataset. The peak area of the most robust ion (known as the quantifier ion) provides a measure of the abundance of the compound in the sample.

Samples were batched into groups with minimal variability in retention time (RT). These batches were subsequently RT corrected to align samples for the duration of the study. Initial deconvolution was performed with Profinder (Agilent). Masshunter Quantitative Analysis software (Agilent) allowed the extracted ions from all 46 samples to be simultaneously inspected for measures of feature consistency and quality. The criteria for the feature inspection and consequential action is described in Table 1.

**Table 1:** Checks for feature consistency and quality

Check	Resolution
Qualifier and quantifier ions were specific for a feature across all samples to prevent misidentification, particularly for those ions selected for calculating peak area	Alternate related ions identified and examined for specificity or feature removed
Ions correlated in RT to within 1.5 s within a single sample and 15 s between samples	Selected ions were removed or added to ensure criteria was met
Correlating peak shape and time alignment between all qualifier and quantifier ions	Ions not possessing matching peak shape were removed
RT extraction window was checked for consistency across all samples	RT window adjusted to ensure peak area was correctly captured in all samples
Ion ratios between selected fragments were flagged if outside ± 15% of the target ion ratio	If ion ratio consistency could not be achieved across multiple samples, the feature was removed.^a^
Feature present in at least 5 samples	If not present in at least 5 samples, feature was removed

^a^This criterion was relaxed for the lowest intensity samples (~ 103 counts on the EIC) as the ratio error increased and in cases where there was an interfering peak on the secondary ion but not with the ion which was selected to have its area extracted

When peaks were initially identified as one feature but were subsequentially split into two features based on the ion correlation in retention time, the criteria (Table 1) were re-run on each new candidate feature. This processing resulted in a set of 25 features, termed molecular features (MFs), suitable for comparison across the sample set. The MFs were matched against the National Institute of Standards and Technology (NIST) standard reference database (2017), a library of over 200,000 compounds, and selected based on the highest match factor (a measure of how well the mass spectrum matches spectra of known standards) to provide a tentative identification. A match factor > 70–80% represents a hit with a good probability of reflecting the true structure or chemical class, but all tentative IDs require structural elucidation analysis or comparison to a true standard to establish the real ID. Tentative IDs are listed in the Supplemental Feature Tables (see Table 2). Features known to originate from analytical equipment such as siloxanes were removed from the list prior to analysis. Resulting features were imported into the Masshunter Quantitative Analysis software (Agilent Technologies) and integrated.

**Table 2:** Feature table with NIST match score

Tentative ID	MF#	NIST match score (%)
Toluene	MF1	60–65
2,4-Dimethyl-1-heptene	MF2	40–50
styrene	MF3	60–70
Heptanal	MF5	75–80
Tricyclo[2.2.2.0(1,4)]octane	MF6	20–40
Butyrolactone	MF7	25–40
3-Carene	MF8	50–55
Nonane, 2-methyl	MF9	30–40
Benzaldehyde	MF10	55–67
Phenol	MF11	55–65
Heptane, 2,2,4,6,6-pentamethyl-	MF12	55–60
Decane	MF13	42–50
Octanal	MF14	55–65
Heptane, 5-ethyl-2,2,3-trimethyl-CC	MF20	< 10
Acetophenone	MF21	10–15
Nonanal	MF22	~ 40
Dodecane/Tetradecane	MF23	40–45
Hexadecane/bromo heptadecane	MF24	45–55
Dodeanal	MF25	40

### Statistical analysis

The relationship between identified MFs and demographic and clinical features was explored using Principle Component Analysis (PCA), an approach to visually inspect the underlying structure of the data.

To investigate whether the peak area of each MF differed between the EIB and NO-EIB patients, we used the Mann–Whitney U test, a two-sample non-parametric test. The analysis included all 25 MFs and did not require the MFs to be normally distributed.

To test whether ICS treatment modified the relation between EIB and the MFs, two-way analysis of variance (two-way ANOVA) was conducted to test for effect modification. A two-way ANOVA was used in the absence of an equivalent non-parametric test. An unadjusted interaction p-value < 0.2 was considered to provide some evidence of an ICS effect modification. Subgroup analysis was then performed on these MFs to investigate whether the peak area differed between the EIB and NO-EIB patients in the ICS-positive and ICS negative stratum. An unadjusted p-value of ≤ 0.05 was considered statistically significant.

Finally, a multivariable approach was taken to see whether a combination of MFs could classify EIB patients better than any single MF. Linear Discriminant Analysis (LDA) with shrinkage calculated from Ledoit-Wolf lemma1 was used to determine how well the MFs can be used to distinguish samples from EIB vs NO-EIB patients. LDA was selected because of the relatively small sample size (46) and the risk of overfitting, which is much higher in non-parametric approaches such as random forest. To further reduce the risk of overfitting, the classification pipeline also included an ANOVA F-test based feature selection step. Only the most significant MFs from the training set were used to construct the LDA model. The optimal M was defined to be the one that maximizes the mean AUC (receiver operating characteristic area under the curve) across all folds generated from repeated stratified K-fold cross-validation. The number of folds was defined such that each left-out set contains at least two samples from the least-represented class. The optimal M was then used in a leave-one-out cross validation to estimate the overall AUC.

A permutation test with five-thousand label permutations was used to assess the statistical significance of the overall AUC. In each permutation, an AUC of the LDA pipeline (with optimized M) was calculated using a leave-one-out cross validation. This generated an empirical null distribution of AUCs, which was then used to calculate the p-value.

## Results

Breath samples were collected and analyzed for 46 patients, 21 with EIB and 25 without EIB (NO-EIB). Initial analysis involved all patients regardless of ICS use. In terms of demographic metrics, the EIB and NO-EIB patients were similar except for FEV_1_ change post-exercise and allergy status as is to be expected (Table 3).

**Table 3:** Patient demographic and clinical metadata

	EIB		non EIB		p-value*	
	ICS negative (n = 10)	Total (n = 21)	ICS negative (n = 13)	Total (n = 25)	ICS negative	Total
Male^a^	8/10	16/21	9/13	19/25	0.66	1.00
ICS use^a^	–	11/21	–	12/25	–	1.00
Allergic rhinitis^a^	8/8	15/17	1/7	6/14	** < 0.01**	**0.02**
Age^b^	10	10	7	7	0.83	0.55
BMI z-score^b^	0.68	0.36	0.16	0.16	0.56	0.83
FEV_1_ pre-exercise^b^	92.8	91.0	89.6	98.3	0.60	0.17
Fall in FEV_1_ (%)^b^	30.4	27.1	5.5	5.5	** < 0.01**	** < 0.01**

Significant results are displayed in bold

^a^Categorical variables shown as fraction of total and percent. Changes in denominator indicate some patients had missing information

^b^Continuous variables shown as median of all patients in each class

^*^p-values calculated by Fisher’s Exact Test for categorical variables and Mann–Whitney U-test for continuous variables

Visual inspection of PCAs didn’t show clear clustering on confounders or clinical labels. Lower and upper breath fractions were analyzed separately to identify MFs discriminating EIB and NO-EIB samples. In both instances, no MFs were significantly different between EIB and NO-EIB samples, with or without correction for multiple comparisons (unadjusted p-values > 0.05). This suggested that there was no evidence of an association between MFs and EIB status in this dataset. Similar results were seen with a multivariate LDA approach, which failed to construct a classifier that performed significantly better than random chance.

### ICS stratified results

When children with asthma were stratified on ICS use, the ICS negative group had 10 EIB patients and 13 NO-EIB patients. These groups were broadly similar on demographic and clinical covariates except for allergies, which was also observed in the unstratified data (Table 3).

### Univariate analysis

Five MFs were found to have an unadjusted p-value < 0.05 for ICS effect modification, two of which, MF23 and MF14, were in both the lower and upper breath samples.

### ICS negative

**Fig. 1:**
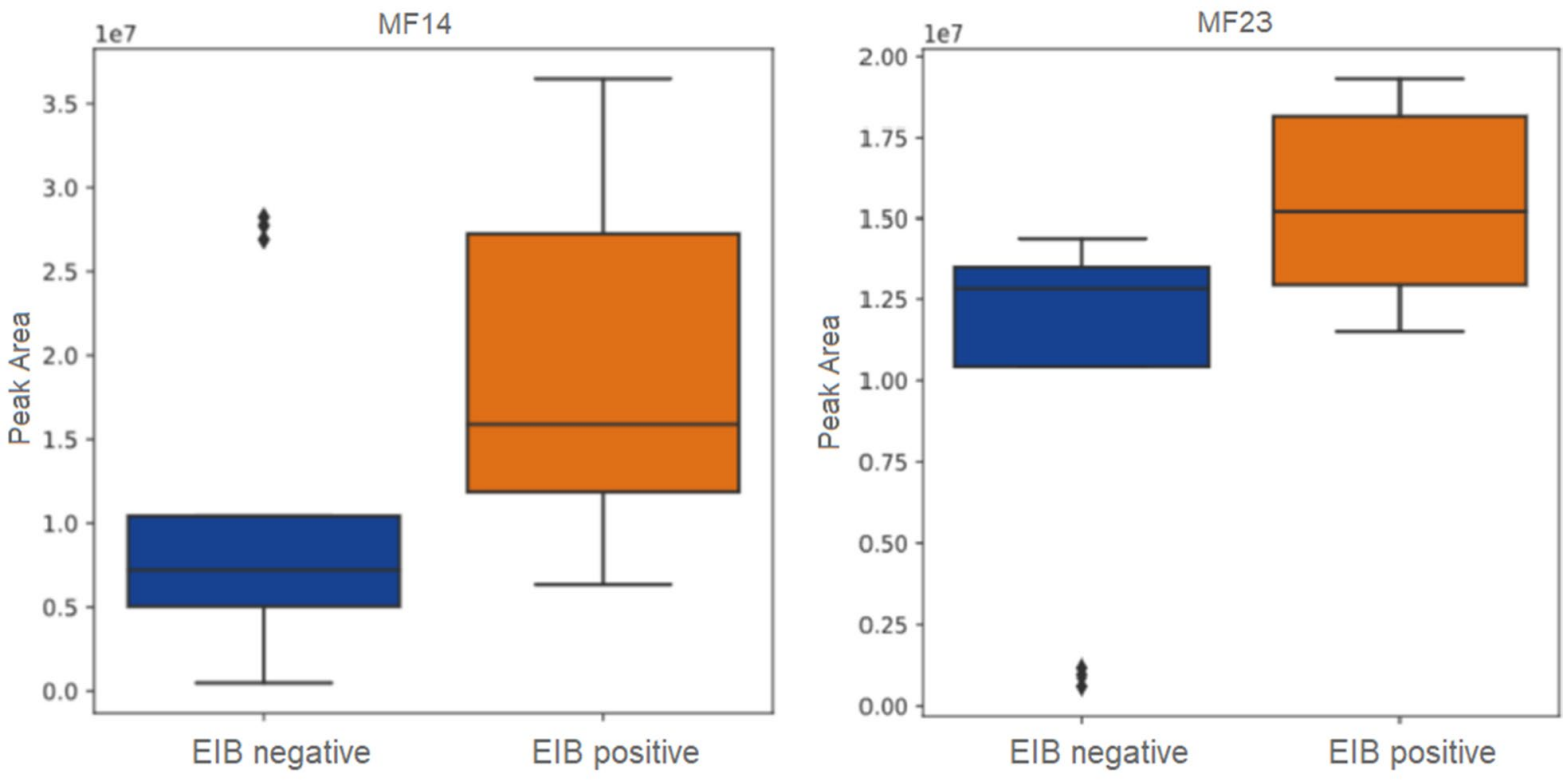
MF peak areas MF14 and MF23. The boxplots visualize the median, interquartile range and minimal and maximal values of the peak area in children without (EIB negative, n = 13) and with (EIB positive, n = 10) exercise induced bronchoconstriction (EIB) within the corticosteroid negative group

In the analysis of the ICS negative samples, in contrast to the unstratified analysis, two MFs, MF23 (p = 0.01) and MF14 (p = 0.03), had an unadjusted p-value < 0.05 for an association with EIB status in the lower breath samples (Fig. 1).

**Fig. 2:**
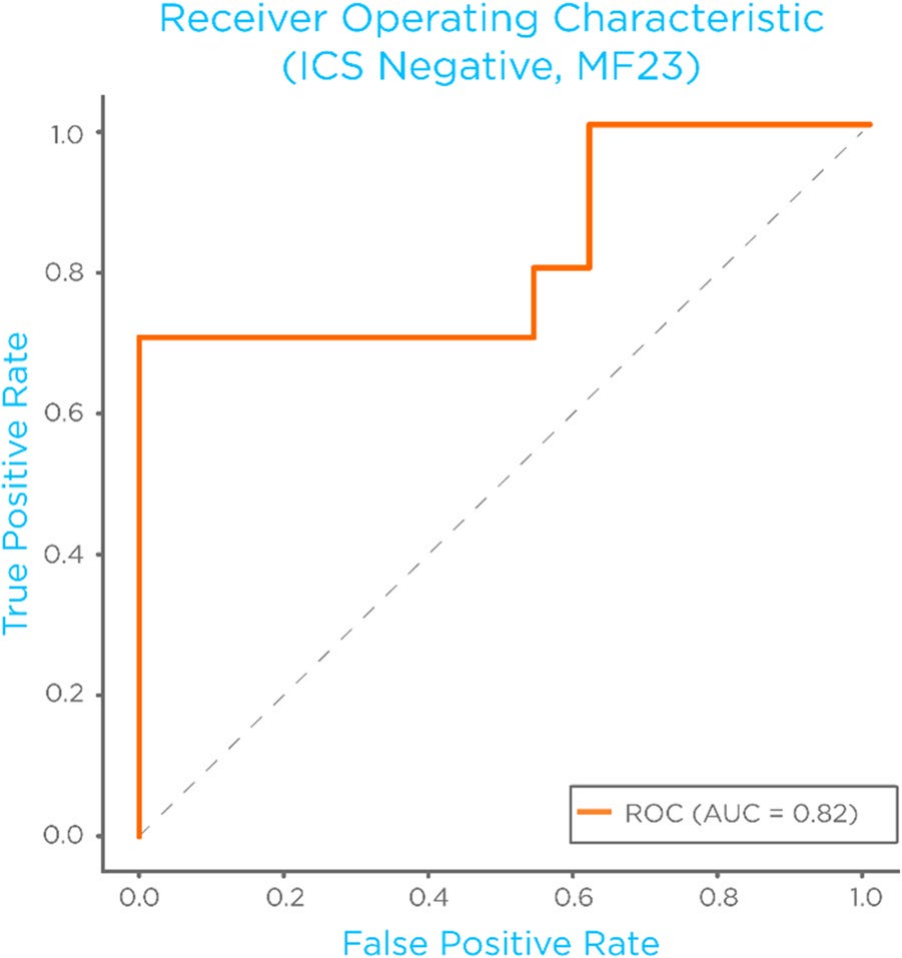
Receiver operating characteristic (ROC)-curve for MF23 for the diagnoses of EIB in the ICS-negative stratum

MF23 also had an unadjusted p-value of 0.03 in the upper breath samples. Both of these MFs had an AUC > 0.70 in both upper and lower breath samples, with MF23 having an AUC = 0.82 in the lower breath samples, the ROC-curve of MF23 for ICS negative subjects can be seen in Fig. 2.

### ICS positive

In the analysis of the ICS positive group, only MF1 had an unadjusted p-value < 0.05 for an association with EIB status. This association between MF1 and EIB status was only observed in upper breath samples. In contrast to the top two MFs for ICS negative samples, MF1 is decreased in EIB patients relative to NO-EIB patients. It can discriminate between EIB patients and NO-EIB patients with an AUC of 0.76. Although a significant association, the NIST match score was low (60–65%), indicating low confidence in the chemical ID of this compound.

## Discussion

This pilot study shows that exhaled breath VOCs in asthmatic children are not significantly different between EIB and NO-EIB. However, in corticosteroid naïve asthmatic children, EIB was found to be associated with increased MF14 and MF23. The tentative identities of these compounds are octanal and dodecane/tetradecane respectively. These compounds have links to lipid metabolism and can be associated with oxidative stress responses. By contrast the corticosteroid positive group showed a reduction in MF1 associated with EIB. MF1 is tentatively identified as toluene, a common component of air pollution, associated with asthma. The exhaled breath VOCs in asthmatic children may therefore differentiate between EIB and NO-EIB but seems to be affected by the use of inhaled corticosteroids.

To our knowledge this is the first study to analyze the association of VOCs from breath with EIB in asthmatic children. We found an effect of ICS on exhaled biomarkers. An unstratified analysis did not find any MFs that significantly differed between the two groups in the available data. The ICS effect modification analysis, which has a plausible pathophysiological rationale, revealed that the effect of EIB status on MF was being modified by ICS treatment. A comparable effect was seen in a study by Chi et al. who found a reduced activity of IL-4 after ICS treatment in asthmatic adults analyzing breath condensate [15].

In the subsequent ICS subgroup analysis, there was small association between compounds prospectively identified as octanal and dodecane/tetradecane and EIB status in the ICS negative stratum. Importantly, these MF’s had unadjusted p-values < 0.05 in both the lower and upper breath samples, suggesting that their associations with EIB status suggesting that this could be a robust association.

EIB is caused by the effect of evaporation of water from the airways during exercise induced hyperpnea [6]. This changes the osmolarity of the fluid layer lining the airway wall [16]. Specifically, the return to isotonic osmolarity after hyperosmolarity leads to release of mediator compounds from mast cells residing in the inflamed airway walls of asthmatics [4, 5]. After an initial prostaglandin release, leukotriene D4 is released contributing to bronchoconstriction following exercise [5]. The production of VOCs is strongly linked to peroxidation of lipids, including lipid mediator compounds [17]. We can therefore speculate that these lipid peroxidation products are the result of breakdown of inflammatory lipid mediator compounds such as prostaglandins in the airways. ICS are powerful anti-inflammatory drugs, which reduce the number and activity of mast cells in the asthmatic airway wall, resulting in less mediator release. The observation that MF23 is suppressed in those treated with ICS fits this hypothesis. In the analysis of the ICS positive stratum, only toluene, had an unadjusted p-value < 0.05 for an association with EIB status. Toluene, a known air pollutant, has previously been associated with asthma and asthma control in children [18]. Indeed we did find a relationship between toluene in breath exudate and the occurrence of EIB, a sign of uncontrolled asthma in asthmatic children [19].

This is a small preliminary study, the scope of the study was partly limited by its integration into an existing study design [10], as such the study sample consisted of a heterogenous group of asthmatic children with regards to for example the allergic rhinitis status and the use of ICS. On the other hand the gender distribution was consistent with the higher prevalence of asthma in boys than girls [20]. Larger validation studies including strategies to reduce confounding will be needed and may produce further results as an expanded sample provides greater statistical robustness. This study can provide guidance what target compounds are relevant to explore in more detail and validate these findings.

This study uses two robust tests making comparison feasible. Bronchoconstriction after an exercise challenge is considered to be indicative of childhood asthma [1]. The laboratory in which the tests were conducted allow for standardized testing throughout the year with a constant air quality (temperature at 9.5–10.5 °C and humidity at 5–7 H_2_0 mg/l air). Furthermore, in order to maximize the chances of finding breath compounds associated with EIB, two breath fraction samples were collected from each patient, upper airway dominated breath and lower airway dominated breath. Previous work has established that the concentration and presence of breath compounds varies over the course of an exhalation [21]. The general hypothesis is that early exhalation contains more exogenous compounds as well as compounds generated by cells in the upper airway while late exhalation contains more systemic, endogenous compounds as well as compounds produced by lower airway cells.

Based on the results of this study, future studies investigating exhaled breath VOCs in asthmatic children should take the use of ICS into account as effect mediator. Moreover, studies in corticosteroid naïve patients are advised in order to investigate in more detail which potential VOC biomarkers may be able to differentiate EIB status in asthmatic children. This may then enable the opportunity for an objective early diagnostic tool for EIB in children who have just been diagnosed with asthma, before start of ICS treatment and without time consuming and expensive EIB evaluation, emphasizing the clinical relevance of such a tool.

## Conclusion

This pilot study has identified several candidate biomarkers that are associated to pediatric EIB, but depend on ICS use and the breath sample fraction collected. As such it provides insight into what breath compounds are of interest and provides potential directions for future studies looking to develop the diagnostic accuracy of such a breath-based test, so that these candidate biomarkers have a potential to enable non-invasive diagnosis of EIB in children.

## Data Availability

The datasets during and/or analysed during the current study available from the corresponding author on reasonable request.
